# MITOCHONDRIAL DNA VARIANT C2639T IS AN APOE4 RESILIENCE FACTOR

**DOI:** 10.1093/geroni/igz038.3417

**Published:** 2019-11-08

**Authors:** Brendan Miller, Su Jeong Kim, Junxiang Wan, Hemal H Mehta, Kelvin Yen, Pinchas Cohen

**Affiliations:** 1 University of Southern California, Los Angeles, California, United States; 2 Leonard Davis School of Gerontology, University of Southern California, Los Angeles, California, United States

## Abstract

The APOE4 allele is the greatest genetic risk factor for sporadic Alzheimer’s disease, yet select APOE4 carriers remain cognitively intact and become centenarians due to unclear reasons. In order to identify resilience genes for APOE4 carriers, we (1) sequenced whole mitochondrial DNA in a centenarian cohort, (2) searched for differentially expressed genes in the temporal cortex of APOE4 carriers, and (3) experimentally simulated the effects of a novel mitochondrial DNA variant that confers APOE4 resilience. The mitochondrial DNA variant, C2639T, is highly enriched in centenarians and APOE4 carriers, which changes the third amino acid of the mitochondrial-derived peptide humanin from proline to serine (humanin P3S). In addition, APOE4 carriers differentially expressed 127 genes in the humanin genetic network that map back to mitochondrial function. Therefore, we experimentally characterized the relationship between humanin, centenarian-enriched humanin P3S, and APOE. We found that humanin is a novel APOE binding partner, humanin P3S binds APOE nearly 15 times greater than wild type humanin, and humanin P3S modifies the APOE4 metabolic profile.

